# Epithelial-to-Mesenchymal Transition in Pancreatic Ductal Adenocarcinoma and Pancreatic Tumor Cell Lines: The Role of Neutrophils and Neutrophil-Derived Elastase

**DOI:** 10.1155/2012/720768

**Published:** 2012-11-20

**Authors:** Thomas Große-Steffen, Thomas Giese, Nathalia Giese, Thomas Longerich, Peter Schirmacher, G. Maria Hänsch, Matthias M. Gaida

**Affiliations:** ^1^Institut für Immunologie, Universität Heidelberg, 69120 Heidelberg, Germany; ^2^Chirurgische Universitätsklinik, Universität Heidelberg, 69120 Heidelberg, Germany; ^3^Pathologisches Institut, Universität Heidelberg, 69120 Heidelberg, Germany

## Abstract

Pancreatic ductal adenocarcinoma (PDAC) is frequently associated with fibrosis and a prominent inflammatory infiltrate in the desmoplastic stroma. Moreover, in PDAC, an epithelial-to-mesenchymal transition (EMT) is observed. To explore a possible connection between the infiltrating cells, particularly the polymorphonuclear neutrophils (PMN) and the tumor cell transition, biopsies of patients with PDAC (*n* = 115) were analysed with regard to PMN infiltration and nuclear expression of **β**-catenin and of ZEB1, well-established indicators of EMT. In biopsies with a dense PMN infiltrate, a nuclear accumulation of **β**-catenin and of ZEB1 was observed. To address the question whether PMN could induce EMT, they were isolated from healthy donors and were cocultivated with pancreatic tumor cells grown as monolayers. Rapid dyshesion of the tumor cells was seen, most likely due to an elastase-mediated degradation of E-cadherin. In parallel, the transcription factor TWIST was upregulated, **β**-catenin translocated into the nucleus, ZEB1 appeared in the nucleus, and keratins were downregulated. EMT was also induced when the tumor cells were grown under conditions preventing attachment to the culture plates. Here, also in the absence of elastase, E-cadherin was downmodulated. PMN as well as prevention of adhesion induced EMT also in liver cancer cell line. In conclusion, PMN via elastase induce EMT *in vitro*, most likely due to the loss of cell-to-cell contact. Because in pancreatic cancers the transition to a mesenchymal phenotype coincides with the PMN infiltrate, a contribution of the inflammatory response to the induction of EMT and—by implication—to tumor progression is possible.

## 1. Introduction

Infiltration of polymorphonuclear neutrophils (PMN) into tumors and the adjacent tissue is observed in numerous cancers. Their role, however, is controversially discussed. On one hand, defence mechanisms are described, on the other, a participation of PMN, specifically of PMN-derived mediators in the progression of the tumor, is likely, and in some cancers, PMN infiltration and the ensuing inflammatory response are linked to poor prognosis (reviewed in [1–3]). We are especially interested in pancreatic ductal adenocarcinoma (PDAC), a particularly aggressive tumor, characterised by early invasion and formation of metastasis [4]. In PDAC, infiltration of PMN was considered to be a rare event, mostly occurring in poorly differentiated tumors with a micropapillary growth pattern [5, 6]. However, an infiltration of PMN into the desmoplastic stroma was seen in the majority of cancer samples [7, 8]. In recent years, it has gained increasing interest, because the infiltration by immune cells may create a proinflammatory microenvironment that affects tumor progression by numerous different mechanisms [9, 10]. 

Another feature of many malignant tumors is the transition of tumor cells to cells with mesenchymal characteristics especially at the invasive front. This so-called “epithelial-to-mesenchymal transition” (EMT) was first described in the context of developmental biology [11], but it also occurs in repair processes, such as wound healing and scarring at sites of persistent inflammation [12]. Typical features of EMT are the change in cell morphology, the loss of cellular polarity, and the downregulation of E-cadherin and keratins. In parallel, mesenchymal markers, such as vimentin or fibronectin [12], are upregulated [13], as is N-cadherin. 

In the context of tumor biology, EMT is of special interest, because acquisition of mesenchymal characteristics is associated with an enhanced migratory capacity, which—by implication—could contribute to invasive growth and formation of metastasis [14]. Data relating EMT to outcome, prognosis, or survival are in part controversial, especially for pancreatic cancers [15, 16], and may relate to problems in reliable detection of EMT in tumor tissues, because EMT is reversible and probably only seen in cells at the invading front [17]. Experimentally, EMT can be induced by numerous cytokines, particularly by transforming growth factor *β*, but also loss of cellular contact, for example, due to degradation of basement membranes, or other modifications of the microenvironment, can induce a genetic program that leads to EMT [12]. Under culture conditions, the process is reversible [18] and occurs within hours.

In the present study, we explored a possible link between the PMN infiltrate and the induction of EMT in pancreatic tumors. As marker for EMT, we analysed the expression of the transcription factor ZEB1 in biopsies of patients with PDAC. ZEB1 is particularly useful because it is required for EMT to occur and because it is expressed in pancreatic tumor cells only, but not in normal pancreatic tissue [15]. We found ZEB1 in the majority of the biopsies, and its expression correlated with the density of the PMN infiltrate. To address the question of a causal relationship between EMT and PMN, we performed *in vitro* experiments and found that PMN via elastase induced EMT in pancreatic tumor cells, and in a hepatocellular carcinoma cell line (HuH7), which was used for comparison, as well.

## 2. Materials and Methods

### 2.1. Biopsy Material and Scoring of PMN Inflammation

PDAC tissue samples were obtained from 115 patients (47 female, 68 male; age range: 39–85 years; mean: 64.9 years; median: 66.0 years) and HCC samples from 39 patients (2 female, 37 male; age range: 47–80 years; mean: 65.2 years; median: 67.0 years). The tissue specimens were formalin fixed and paraffin embedded, and following the haematoxylin and eosin (H&E) staining, the diagnosis of PDAC, respectively, HCC was established according to the criteria recommended by the World Health Organization (2010) [19]. For the PDAC samples, the tumor stage was established according the criteria of UICC (2009). Of 104 PDAC patients survival data were available. 61 patients died from PDAC within 25–1187 days after the operation (mean: 427 days, median: 347 days), 37 patients were alive after a follow-up period of 15–1044 days (mean: 551 days, median: 663 days), and 6 patients died on noncancer related causes and were excluded from the study. In accordance to a previously published system [8, 20], the infiltration of neutrophils was scored as absent (score: 0), intermediate (score: 1), or severe (score: 2), depending on the density of infiltrated neutrophils. Neutrophils within the tumor as well as cells in the adjacent desmoplastic stroma were counted, but not neutrophils within blood vessels or localised abscesses. The study was approved by the Ethic Committee of the University of Heidelberg and written informed consent was obtained from the patients.

### 2.2. Immunohistology

Paraffin-embedded tissue sections (4 *μ*m) were analysed using the avidin-biotin complex method as previously described [21]. Prior to antibody incubation, heat pretreatment in an antigen retrieval solution (DAKO Cytomation, Hamburg, Germany; pH 9.0 for neutrophil elastase), respectively, using citrate buffer (pH 6.1 for *β*-catenin and ZEB1) was performed. Primary antibodies included a mouse monoclonal antibody to neutrophil elastase (American Diagnostics, Pfungstadt, Germany, diluted 1 : 25), a mouse monoclonal to *β*-catenin (BD, Pharmingen, Heidelberg, Germany; 1 : 500), and a monoclonal anti-ZEB1 (Abcam, Cambridge, UK; diluted 1 : 40 for PDAC and 1 : 200 for HCC). PMN were visualised using naphtol-ASD-chloroacetate esterase (Sigma, München, Germany) and were counted in 10 high power fields (400x), in the tumor, in the vicinity of the tumor cells, and in the desmoplastic tumor stroma as well. Areas with abscesses, necrosis, and foreign body reaction (bile leakage, suture material), accompanied by a PMN reaction, as well as PMN in blood vessels, were excluded from the evaluation. Of the 10 counted fields, the mean value was calculated and used for scoring. Results of ZEB1 and *β*-catenin expression were quantified according to the well-established “Allred-Score” [22], a summation of the percentage of tumor cells expressing the respective antigen and the intensity of its expression. These findings were correlated with the density of the PMN infiltrate.

### 2.3. Statistical Analysis

Correlation of ZEB1 expression with the PMN infiltrate was calculated using Mann-Whitney *U*-test (two-tailed) and Spearman's-Rho analysis. Clinical and pathological parameters were compared with Spearman's-Rho analysis, the survival data with log-rank test. The statistical analyses were carried out with the SPSS software version 18.0 for Windows (SPSS, Chicago, USA). Graphs were made using OriginPro7.5 software (Additive Software, Friedrichsdorf, Germany). 

### 2.4. Isolation of Polymorphonuclear Neutrophils (PMN)

Peripheral blood from healthy human volunteers was obtained by puncture of peripheral veins and collected in heparin-NH_4_ coated tubes (Sarstedt, Nürnbrecht, Germany). PMN were isolated by centrifugation on PolymorphPrep (Axis-Shield PoC AS, Oslo, Norway) which yielded an 85% to 95% pure PMN population. The PMN were suspended in Hanks balanced salt solution (HBSS) and used within 1 h. Informed consent was obtained from the donors and the study was approved by the local ethics committee.

### 2.5. Culture of the Tumor Cell Lines

The human pancreatic cancer cell line T3 M4 (provided by the European Pancreas Center, Heidelberg, Germany) and the liver cancer cell line HuH7 (provided by the Institute of Pathology, Heidelberg, Germany) were grown in RPMI-1640, respectively, DMEM medium containing 10% fetal bovine serum (FBS), 100 U/mL penicillin and 100 *μ*g/mL penicillin-streptomycin, and 1% L-Glutamine (Invitrogen, Karlsruhe, Germany) and were incubated at 37°C in a 5% CO_2_ humidified atmosphere.

### 2.6. Coculture of Tumor Cells with PMN

T3 M4 or HuH7 (1 × 10^6^ in 2 mL) were cultivated in 6 well plates (Nunc, Roskilde, Denmark) for 24 h when they reached confluency. Then, isolated PMN (3 × 10^6^) were added and culturing was continued (37°C in a 5% CO_2_ humidified atmosphere). Dyshesion was determined after various time intervals by quantifying the cell-depleted areas (see below). Alternatively, neutrophil elastase (Calbiochem, Darmstadt, Germany) (3 *μ*g/mL) (≥20 units/mg) was added to serum-free medium. 

Furthermore, up to 1 × 10^7^ PMN with 15 *μ*g/mL *α*-1-antitrypsin (Sigma), 50 nmol/mL of the neutrophil elastase inhibitor IV (Calbiochem), or 50 *μ*mol/mL of the elastase substrate (N-(Methoxysuccinyl)-L-alanyl-L-alanyl-L-prolyl-L-valine chloromethyl-ketone) (Sigma) were added in serum-free medium.

To exclude potential cytotoxic effects of PMN on tumor cells, the tumor cells were preloaded for 30 min with 5 nM calcein (Sigma), and then incubated with PMN for different time points up to 24 h. 

### 2.7. Culture of T3 M4 and HuH7 in Hydrocell Culture Plates

Hydrocell culture plates (Nunc) were used, since they prevent the attachment of the tumor cells, and hence the formation of a monolayer. T3 M4 or HuH7 cells in 5 × 10^6^/2 mL were grown from 1 h to 72 h, in RPMI, respectively, DMEM, and containing 10% FBS, 100 *μ*g/mL penicillin-streptomycin, and 1% L-Glutamine.

### 2.8. Elastase Treatment of T3 M4 and HuH7

T3 M4 and HuH7 were seeded in a concentration of 5 × 10^5^ cells/2 mL in a 24-well culture plate (Nunc, Roskilde, Denmark) for 24 h. Then, the medium was replaced by serum-free medium containing 3–9 *μ*g/mL neutrophil elastase (Calbiochem, Darmstadt, Germany). After various times (1 h to 72 h), the cells were harvested. After 24 h, the medium was replaced by FBS-containing medium. 

### 2.9. Quantification of Dyshesion

After various times, the cells were fixed in 100% ice-cold methanol for 1 min, then digital photographs of 5 representative areas were taken (Leica, Heerbrugg, Switzerland) at the magnification of 10-fold of 5 independent experimental subsets. The cell free areas were quantified using ImageJ-software (open source). The “free” areas were digitally marked and quantified, following the calculation of the ratio: free area/area of the whole tumor cell layer. 

### 2.10. Cytofluorometry

For cytofluorometry, the tumor cells were harvested using ice-cold saline and a cell scraper. The PE-labelled monoclonal mouse anti-E-cadherin (Biolegends, San Diego, USA) or PE-labelled IgG1/IgG2 (BD, San Jose, USA), as isotypic control was used. Following incubation with the respective antibodies (20 min, room temperature), cells were analysed by FacsCalibur using CellquestPro 3.0.1 as software (Becton and Dickinson, Heidelberg, Germany). Results are expressed as mean fluorescence intensity (mean of all) in the appropriate gate. Ten thousand cells were counted. For intracellular staining, cells were permeabilized using 75% ice-cold methanol/25% acetone (v/v). Intracellular staining of keratins was performed using mouse anti-KL1 (Immunotech, Marseille, France) with a FITC-conjugated secondary goat-anti-mouse antibody (Jackson Immuno Res., Suffolk, UK). To exclude the PMN population in the cocultivation experiment, a PE-labelled mouse-anti-CD66b (Miltenyi Biotech, Bergisch Gladbach, Germany) was used.

### 2.11. Detection of E-Cadherin by ELISA

Soluble E-cadherin in cell culture supernatants was determined using a commercially available ELISA kit (Quantikine ELISA Kit, R&D Systems, Darmstadt, Germany) according to the manufacturer's instructions. All samples were at least measured in duplicate.

### 2.12. Quantitative Real-Time Polymerase Chain Reaction

mRNA and cDNA preparation kits were purchased from Roche Applied Sciences (Mannheim, Germany). For the preparation of mRNA, the automated MagNA Pure LCinstrument and corresponding isolation kit I (for cells) was used. 5 × 10^5^ cells/2 mL T3 M4 and HuH7 cells after elastase treatment or hydrocell culture were used. All the primers were obtained from Search-LC (Heidelberg, Germany). cDNA was prepared by using a first strand cDNA Synthesis kit. Subsequently, PCR was performed with the LightCycler-Fast Start DNASYBR Green kit. Cyclophilin-B (CPB) was used as a housekeeping gene to normalize the expression of specific transcripts of Twist, Snail, and keratin 19 (K19).

### 2.13. Protein Isolation, SDS-PAGE, and Western Blot

Proteins of the subcellular components (membrane, cytoplasm, nucleus, cytoskeleton) from 3 × 10^6^ T3 M4 or HuH7 cells with or without treatment of neutrophil elastase (3 *μ*g/Ml for 2 h) were isolated using the ProteoExtract-kit (Calbiochem/Merck, Darmstadt, Germany), according to the manufacturer's recommendation. Protein samples were boiled for 10 min at 95°C and separated by SDS-polyacrylamide gel (7%) electrophoresis. After blotting to a nitrocellulose transfer membrane (Whatman, Dassel, Germany), the following antibodies were used: a mouse monoclonal antibody to *β*-catenin (BD Biosciences, New Jersey, USA), or to ZEB1 (Abcam, Cambridge, UK), KL-1 (Immunotech, Marseille, France), anti-mouse IgG-POX (Jackson Immunoresearch, Pennsylvania, USA); a rabbit antibody to human E-cadherin (Santa Cruz, Biotech, Santa Cruz, Ca, USA) and anti-rabbit IgG POX (Jackson Immunoresearch). To control for equal loading, *β*-actin (cytoplasmatic extracts) or p84 (nuclear extracts) was determined using antiactin or anti-p84, respectively (both obtained from Abcam, Cambridge, UK). For signal detection, Amersham ECL plus Western Blotting Detection System (GE Healthcare, Munich, Germany) was used. 

## 3. Results

### 3.1. PMN Infiltrates and EMT in Tissue of Pancreatic and Hepatocellular Carcinoma. 

**Figure 1:**
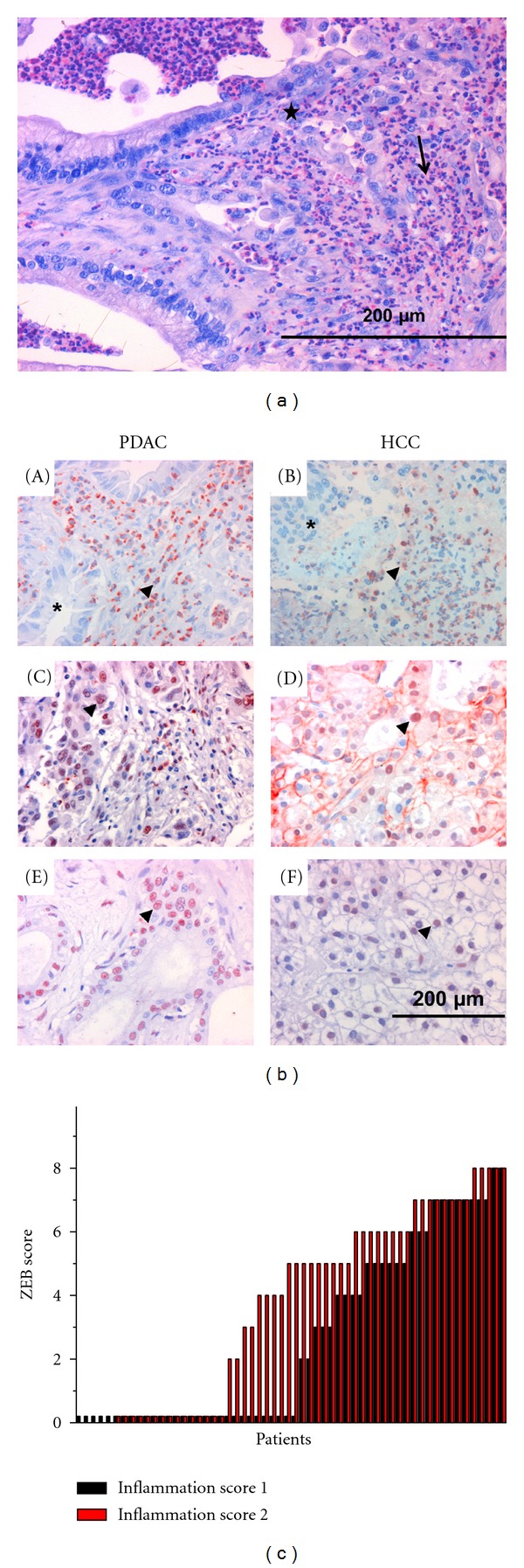
(a) PMN stained with NASDCL (dark purple) are shown in a specimen of a PDAC sample (magnification ×200). (b) (A) The tumor cells forming a glandular growth pattern (asterisk) were surrounded by elastase positive PMN (arrow). In (B), a solid structured HCC specimen is shown (asterisk) infiltrated by elastase positive PMN (arrow). Nuclear accumulation of *β*-catenin is seen in the PDAC sample (C) and the HCC sample (D), as is nuclear accumulation of ZEB1 in a PDAC (E) and a HCC biopsy (F) (examples marked by arrow heads). (c) The intensity of ZEB1 was determined (Allred score) in the PDAC specimen and correlated to the PMN infiltrate, either scored as intermediate (black) or severe (red). Each bar represents one specimen.

The inflammatory infiltrate was analysed in biopsies of 115 PDAC samples. PMN, identified by the expression of NASDCL or elastase, were found in the majority of samples (111/115) (Figures 1(a) and 1(b)). According to density of the PMN infiltrate, the following groups were formed: no infiltrate (score 0; 4/115), intermediate (score 1; 58/115), and severe infiltrate (score 2; 53/115). As markers for EMT, the nuclear accumulation of *β*-catenin was assessed as was expression of ZEB1 (examples in Figure 1(b)). Only in 8 of the 115 samples (7%), nuclear *β*-catenin was detected. Of note, 7 of these sample had high PMN infiltrate (score 2). ZEB1 was found in 71/115 (62%) biopsies (data summarised in Table 1). When using the Allred score for quantification of ZEB1, high scores coincided with severe PMN infiltration (Figure 1(c)). The distribution was marginally significant according to the Spearman rho test (*P* = 0.027). Using the same test, neither the expression of ZEB1 nor the density of the neutrophil infiltrate correlated with the TNM status, histological grading, or patients' survival. 

In 31 of 39 HCC samples, PMN were found and intensity was scored as described for the PDAC samples: score 0 (8/39), score 1 (20/39), and score 2 (11/39). Nuclear accumulation of *β*-catenin was noted in 9/39 samples (31%), 12/39 cases showed a nuclear ZEB1 expression (Table 1). Neither the *β*-catenin nor the ZEB1 expression correlated with the density of the infiltrated PMN.

### 3.2. Neutrophil Elastase Cleaves E-Cadherin and Induces EMT in Cancer Cells

**Figure 2:**
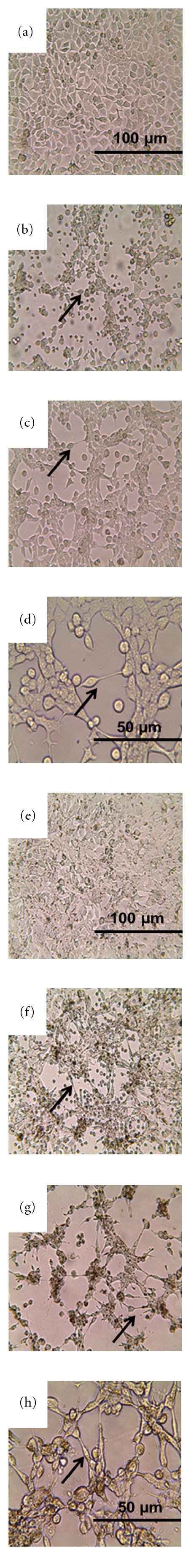
PMN or PMN-elastase induce dyshesion of tumor cells and loss of E-cadherin: pancreatic cancer cells (T3 M4; upper panel) and liver cancer cells (HuH7, lower panel) were seeded as monolayers (a and e), and then incubated with PMN (b and f), or PMN-elastase (c and g) for three hours (magnification ×400; d, h are zooms thereof). Dyshesion of cells was observed, and cells assumed a spindle shaped form (in d and h marked by an arrow).

**Figure 3:**
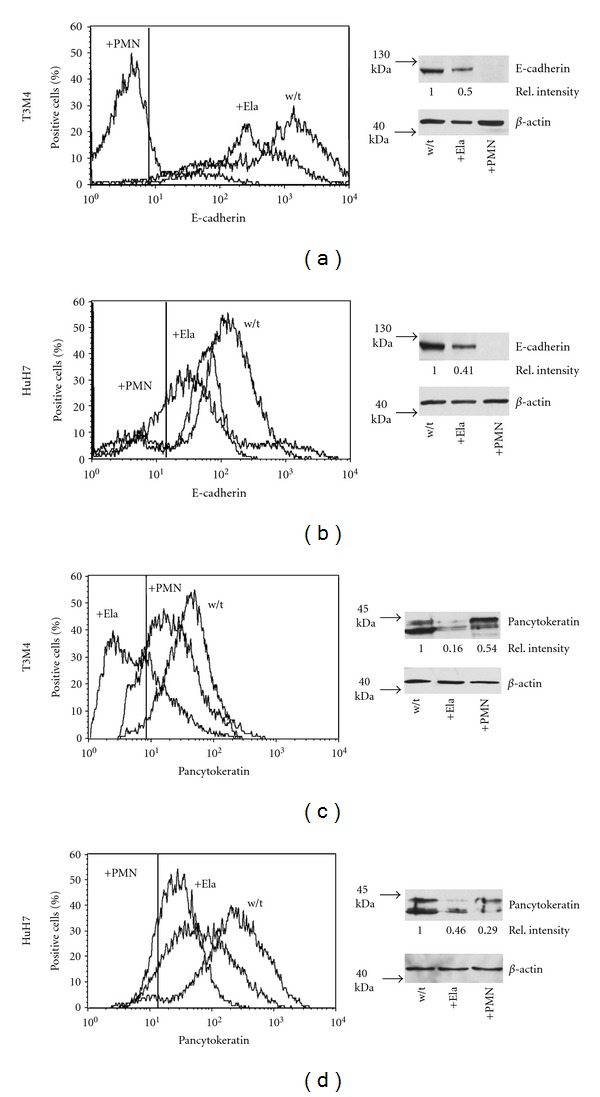
(a and b): Surface expression of E-cadherin on T3 M4 or HuH7 was determined by cytofluorometry (left panels). The manually dispersed cells without treatment (w/t), cells co-cultivated with PMN (PMN) or PMN-derived elastase (Ela) for 3 h are shown (the vertical line shows the position of the end of the IgG-isotype control). On the right panels, the respective Western blots are shown. (c and d): Expression of keratins was determined by cytofluorometry with permeabilised cells (untreated, or co-cultivated with PMN-elastase or PMN for 24 h or by western blotting).

To assess a potential relationship of PMN infiltrates and EMT, monolayers of T3 M4 or HuH7 were incubated with isolated PMN. Within 3 h, the tumor cells assumed a fibroblast-like appearance layer and areas devoid of cells were seen (Figure 2). The effect could be reproduced with isolated PMN elastase and was blocked by the selective elastase inhibitor IV. The cells remained viable, and free-floating cells were rarely detectable. In response to either PMN or isolated elastase, loss of E-cadherin from the surface was seen in both cell lines (Figure 3). In parallel, E-cadherin was detected in cell culture supernatants of T3 M4 cells by ELISA. In untreated cells, 18.7 pg/mL were detected compared to 198.3 pg/mL in the elastase-treated cells (mean of three experiments performed in duplicates; *P* = 0.017 calculated by ANOVA). 

**Figure 4:**
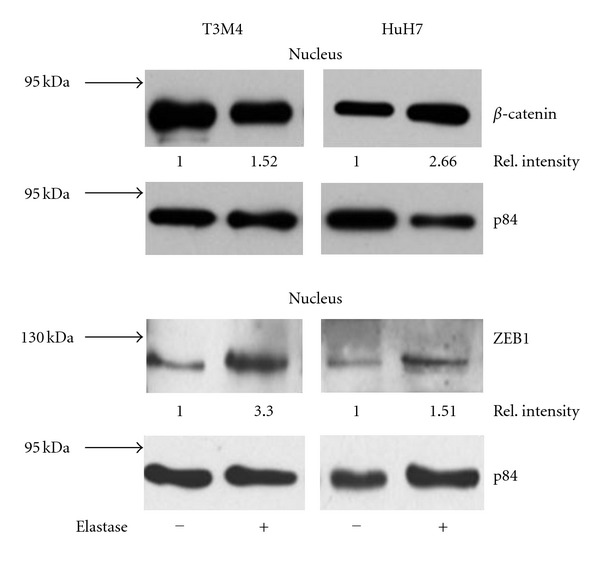
Identification of *β*-catenin and of ZEB1 in the nucleus. The cancer cells were treated with either PMN-elastase (3 h) or left untreated. Then *β*-catenin and ZEB1 were determined in the nucleus (p84 was used as loading control).

In response to elastase or PMN, the keratin expression declined (Figure 3; data are summarized in Table 2), and the abundance of keratin-specific mRNA declined (on average by 60% at 72 h in T3 M4; mean of two independent experiments). Within 3 h after exposure to either PMN or to elastase, ZEB1 was detected in nucleus, as was *β*-catenin, the latter, however, only marginally in T3 M4 (Figure 4). These data collectively indicate an epithelial-to-mesenchymal transition (EMT). In line with EMT, an increase in mRNA of the pertinent transcription factors TWIST and SNAIL was seen: within 24 to 48 h, TWIST increased by 180% in T3 M4 and SNAIL by 150%. In HuH7, TWIST transcripts were not detectable, neither before nor after exposure to elastase. SNAIL was found to be increased by 190% (all values are the mean of two independent experiments done as duplicates). 

The transition was reversible. After prolonged incubation, in the absence of elastase or PMN, respectively, the monolayers were restored, and the cells expressed again E-cadherin (data not shown). 

### 3.3. Prevention of Cellular Adhesion Induced Loss of E-Cadherin and EMT in Cancer Cells

**Figure 5:**
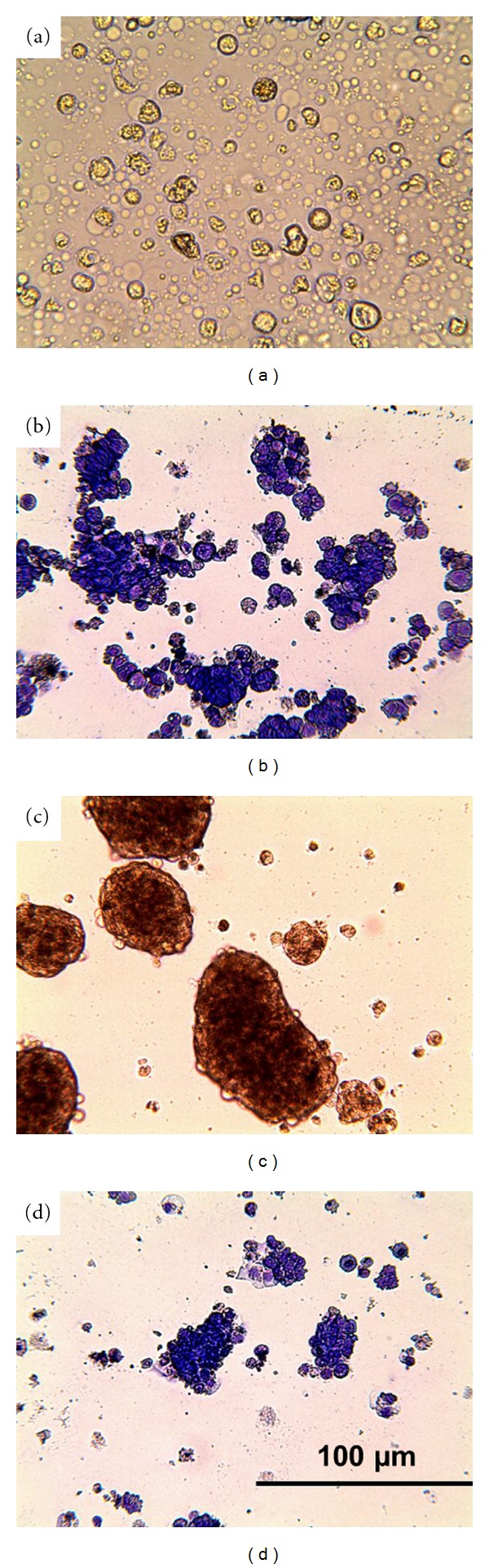
Cells, T3 M4 (upper panel) and HuH7 (lower panel), were grown on hydrocell plates for 24 h (a and c). By light microscopy, formation of cell clusters was seen (b and d show the Pappenheim staining) (magnification ×400).

**Figure 6:**
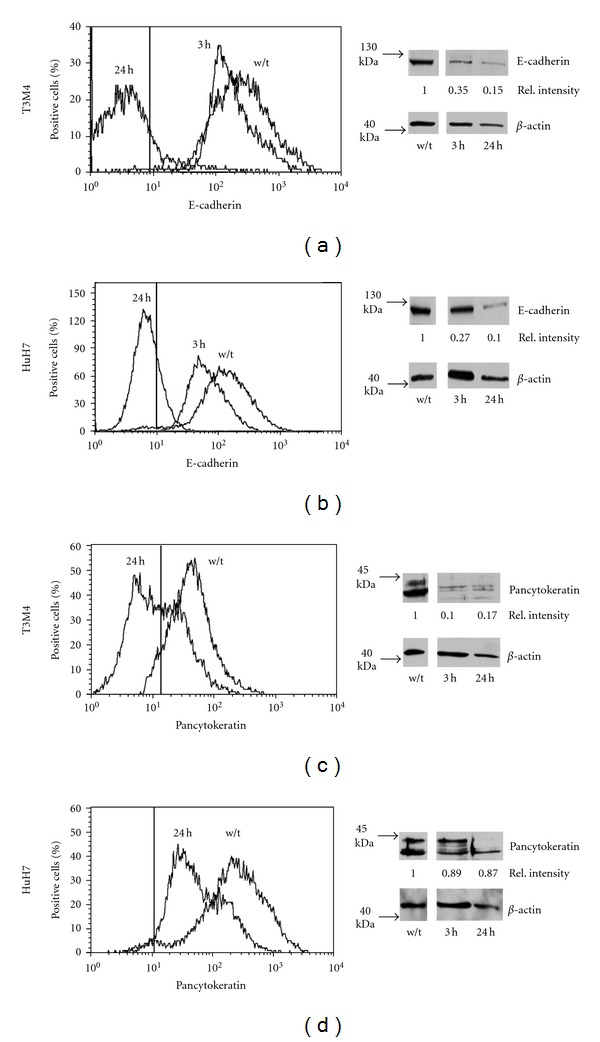
Under nonadherent conditions (grown on hydrocell) for either 3 h or 24 h, E-cadherin is lost, as was keratin measured intracellularly (24 h). On the right panels the Western blots are shown.

**Figure 7:**
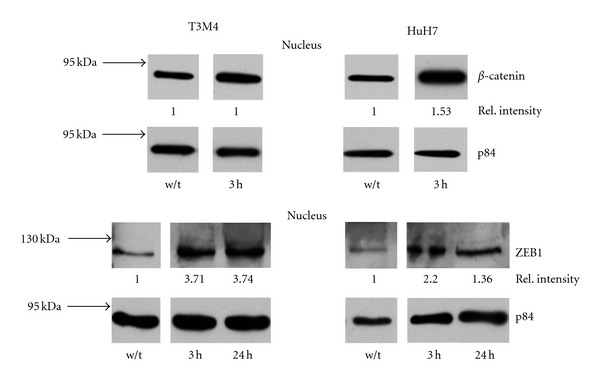
Identification of *β*-catenin and of ZEB1 in the nucleus. The cancer cells were either grown on hydrocell (HC) for three hours or in regular culture plates. Then *β*-catenin and ZEB1 were determined in the nucleus.

In a second set of experiments, T3 M4 and HuH7 were grown in “hydrocell” culture dishes. The surfaces of the culture dishes are modified to prevent adhesion of cells to the device. The cells remained floating, but attached to each other forming tubular or spherical structures (Figure 5); they, however, remained viable. As early as three hours after starting the culture, the E-cadherin expression declined. Prolonged culture resulted in a downmodulation of keratins (Figure 6; Table 3) and by a decline of the keratin specific mRNA, the latter particularly obvious in HuH7 cells, where expression declined to 40% after 24 to 48 h. In HuH7, *β*-catenin translocated to the nucleus, where also ZEB1 was detected (Figure 7). Of the transcription factors, SNAIL increased on average by 300% in both, T3 M4 and HuH7, as early as 3 hours after transferring the cells to the hydrocell plates. 

## 4. Discussion

Pancreatic ductal adenocarcinoma (PDAC) is a particularly aggressive tumor with early dissemination of the tumor cells and invasive growth behavior. PDAC is associated with a pronounced fibrotic reaction, resulting in a so-called desmoplastic stroma [23]. The latter is thought to contribute to disease progression, by mediating the resistance towards chemotherapeutic agents and providing an inflammatory microenvironment that could modulate tumor progression and local defence mechanisms as well [24–26]. Of note, desmoplastic stroma is known to harbour immune cells including PMN [7, 8]. In the present study with biopsies of PDAC patients, we could demonstrate infiltration of PMN into the tumor and into the desmoplastic stroma in the majority of patients. The density of the infiltrate varied among the patients, but it did not correlate with clinical parameters such as TNM status, metastasis, or survival. Studies by other authors showed an association of the intensity of the PMN infiltrate with a poor histological differentiation and a poor prognosis for PDAC and cancers of the periampullary region [5, 6]. In other cancers, such as gastric adenocarcinoma, the density of infiltrating PMN correlated with the metastases and survival, and PMN infiltration was suggested as an independent prognostic marker [27]. A possible explanation is that the majority of the PDAC patients had similar TNM status and that the survival rate in the majority of PDAC is equally poor making a distinction impossible. Tumor infiltrating PMN were also found in the HCC samples, however, less pronounced compared to the PDAC tissue. 

Notably, the PMN infiltrate correlated with expression of the tumor cells of ZEB1. ZEB1 is a transcription factor required for the induction of EMT, and the expression ZEB1 protein is a suitable marker for EMT, because ZEB1 is not expressed in normal pancreatic tissue, but strongly in pancreatic carcinoma. In our study, we found ZEB1 in the majority of cases. Its expression varied among the patients, but not in any correlation to clinical parameters such as TNM status, grading, or survival. A correlation of other EMT markers with clinical parameters was reported in numerous cancers, particularly breast cancer [28], but also in tumors of the gastrointestinal tract [29]. For pancreatic cancer, the data are not yet conclusive [15]. Numerous studies reported evidence for EMT in correlation with survival or metastasis [30, 31], while others did not [32]. ZEB1 expression was studied so far only in a small number of PDAC patients, where a reduced expression was found in patients following a complete resection with no tumor recurrence [33], in line with the fact that ZEB1 is expressed by poorly differentiated tumors. Together with the data from the literature our present study confirms that EMT occurs in PDAC, and in HCC as well, the latter determined by nuclear expression of ZEB1 in the biopsies. 

That ZEB1 expression correlated with the density of the PMN infiltrate leads to the presumption that PMN or PMN-derived entities could participate in the induction of EMT. This presumption was supported by the fact that nuclear expression of *β*-catenin, although a rare event, was seen predominantly in PDAC with severe PMN infiltrate (in 6 out of 7 cases). To assess a possible relationship between PMN and induction of EMT, cells of a pancreatic cell line grown as a monolayer, were co-cultivated with PMN of healthy donors. Within hours, dyshesion of the tumor cell monolayer was observed, a drastic shape change of the cells towards spindle-like appearance, and a loss of E-cadherin from the tumor cell surface. Subsequent experiments revealed that the PMN-derived elastase mediated the loss of E-cadherin. That E-cadherin is a target for elastase was shown previously by others in a rat pancreatitis model and for pancreatic tumor cells as well [34]. 

Following treatment with PMN or PMN-derived elastase, the tumor cells not only lost contact with each other, they also underwent EMT, as determined by established markers for EMT, including loss of keratins, translocation into the nucleus of *β*-catenin, and upregulation of ZEB1. As expected, essentially similar data were obtained for a liver tumor cell line. 

We then asked the question how PMN or more specifically the PMN-derived elastase induces EMT. Induction of EMT *in vitro* is especially well studied in response to cytokines, particularly to transforming growth factor *β*, and also the pertinent transcription factors Snail, Slug, Twist, and ZEB1 and ZEB2 have been identified [12]. Moreover, in the majority of pancreatic tumors, K-ras, which is crucial for EMT, is constitutively activated due to oncogenic mutations, which in turn might favour the transition process [35].

Cultivation of the tumor cells in special culture dishes preventing adhesion also induced EMT. E-cadherin was downregulated under these conditions, compatible with the interpretation that the inability of the cells to adhere causes the downregulation of E-cadherin rather than cleavage by elastase. Hence, loss of cell-to-cell contact might be sufficient to induce EMT. This interpretation is in line with data in the literature, describing induction of EMT following degradation of basement membranes and disruption of the cell-to-membrane contact [12, 18], or a disruption cell-to-cell contacts [36–38] or when epithelial cells are grown as spheroids rather than as monolayers [39]. Other signaling events mediated by elastase, however, cannot be ruled out, such as internalization of the cleaved E-cadherin. Recent data by McGarry Houghton's group [40], showing uptake by tumor cells of PMN-derived elastase, open up new vistas for elastase-dependent signals. 

In summary, we provided evidence that neutrophil-derived elastase cleaves E-cadherin on PDAC- and HCC-cell lines. This eventually initiates a genetic program, resulting in the loss of epithelial markers and the gain of mesenchymal markers. Also, in PDAC tissue samples infiltrating neutrophils correlated with tumor cell expression of the EMT marker ZEB1. So far, we can only speculate on the functional consequences that pancreatic tumor cells having undergone EMT are more resistant towards chemotherapeutic agents [25] and—as we have recently shown for pancreatic tumor cell lines—following EMT, the capacity for migration and invasion is enhanced (own unpublished observation). Thus, our data support the notion that infiltration of PMN may support invasive growth and tumor progression of PDAC via induction of EMT.

## Figures and Tables

**Table 1:** PMN infiltrates, *β*-catenin, and ZEB1 expression in the patients.

	PDAC (*n* = 115)	HCC (*n* = 39)
PMN infiltration	(i) Score 0: 4 (4%)	(i) Score 0: 8 (21%)
	(ii) Score 1: 58 (50%)	(ii) Score 1: 20 (51%)
	(iii) Score 2: 53 (46%)	(iii) Score 2: 11 (28%)

*β*-catenin expression (Allred score)	(i) Score 0: 107 (93%)	(i) Score 0: 30 (77%)
	(ii) Score 2: 2 (2%)	(ii) Score 2: 0
	(iii) Score 3: 0	(iii) Score 3: 2 (5%)
	(iv) Score 4: 5 (4%)	(iv) Score 4: 3 (7%)
	(v) Score 5: 1 (1%)	(v) Score 5: 2 (5%)
	(vi) Score 6: 0	(vi) Score 6: 1 (3%)
	(vii) Score 7: 0	(vii) Score 7: 1 (3%)
	(viii) Score 8: 0	(viii) Score 8: 0

ZEB1 expression (Allred score)	(i) Score 0: 44 (38%)	(i) Score 0: 27 (69%)
	(ii) Score 2: 5 (4%)	(ii) Score 2: 1 (3%)
	(iii) Score 3: 5 (4%)	(iii) Score 3: 6 (15%)
	(iv) Score 4: 10 (9%)	(iv) Score 4: 4 (10%)
	(v) Score 5: 16 (14%)	(v) Score 5: 0
	(vi) Score 6: 12 (11%)	(vi) Score 6: 2 (5%)
	(vii) Score 7: 16 (14%)	(vii) Score 7: 1 (3%)
	(viii) Score 8: 7 (6%)	(viii) Score 8: 0

**Table 2:** PMN and PMN-elastase cause dyshesion, loss of E-cadherin expression, and downmodulation of keratin in cancer cells.

*n* = 3	T3M4		HuH7	
Cultivated with	PMN	PMN elastase	PMN	PMN elastase
Dyshesion*	47,19% (±2,30)	38,86% (±7,33)	37,05% (±1,91)	46,33% (±3,84)
E-cadherin expression**	53,81 (±20,22)	78,76% (±19,64)	22,61% (±5,55)	54,12% (±23,04)
Keratin expression	36,78% (±20,13)	31,85% (±20,18)	17,57% (±9,56)	37,20% (±28,77)

*Measured as area devoid of cells in relation (%) of the total cell area.

**Measured as mean fluorescence intensity (MFI) in relation to the MFI of untreated cells which were set as 100 %.

**Table 3:** Cultivation of T3M4 or HuH7 on hydrocell dishes causes loss of E-cadherin and downmodulation of keratin.

*n* = 3	T3M4	HuH7
	Culture for 24 h	
E-cadherin expression**	16,74% (±12,65)	28,22% (±22,08)
Keratin expression**	63,33% (±26,91)	27,79% (±15,47)

**Measured as mean fluorescence intensity (MFI) in relation to the MFI of untreated cells which were set as 100%.
